# Miner’s Wrist

**Published:** 1878-07

**Authors:** 


### Selections.

---

Miner's Wrist.—After a somewhat extended search through the literature of medicine, both standard and floating, I am unable to find any direct allusion to the condition about to be described, and which I have taken the liberty to call miner's wrist. I have chosen this heading, not because the affection is exclusively confined to miners, but because it is more noticeable among them than elsewhere, and more particularly on account of its brevity.

The affection consists of an inflammation of the muscular structure, or its sheath, or both, of the extensor ossis metacarpi and of the primi internoddi pollicis. It is characterized by swelling, contraction and progressively increasing painfulness of the muscles involved, which is much aggravated by motion, and especially by attempts at supination or pronation of the hand. Crepitation of a marked and peculiar character comes on sooner or later, and is elicited by the motions aforesaid or by ab– and adduction of the metacarpal bone of the thumb.

Prof. Gross, in his System of Surgery, speaks of an analogous condition under the head of "Painful Crepitation," but fails to make any allusion to these particular cases, or indeed to mention these muscles. He very aptly illustrates the crepitus as being not unlike the creaking of snow under foot on a very cold night, or to that produced by rubbing grains of coarse starch between the fingers.

It generally attacks the miner a short time after resuming work succeeding a period of idleness. There is seldom much discoloration of the integument, but the swelling is sometimes very marked, making a roll of considerable magnitude, which passes diagonally across the lower third of the forearm on its radial side from above and somewhat outside the radius downwards and upwards.

The pathology of the case is not exactly known; there never having been an opportunity to verify by *post-mortem*, but there can be but little doubt that a thecal inflammation exists with inflammatory exudation and roughening of apposed surfaces, wherein is derived the crepitus. The person affected generally quits work and applies energetic measures of treatment. Some few, however, have the fortitude to continue. Both alike recover. The prognosis is good. I have never known a case to assume a subacute or chronic form, to leave weakness or irritability of the parts affected, or to entail a susceptibility to a return of the affection. There seems to be no relationship between it and rheumatism, gout, syphilis or any known dyscrasia or specific malady.

Treatment: rest, a nicely adjusted bandage, soap liniment. The time required to affect a cure is from seven to fourteen days.

It may be questioned why miners are more obnoxious to this disease than other working men. It is simply because of the peculiar motion in handling the pick, and more especially in the process known technically as "bearing in," when he cuts his way under the face of the coal some five or six feet, and much of the time lies on his side and swings the pick at evident disadvantage. Other laborers, however, with the pick, ax or hammer, are also occasionally affected, and I am informed by Dr. Halderman, physician at the Ohio penitentiary, that it is not infrequent in some of the workshops of the institution. I am informed by my brother, Dr. Charles F. Gilliam, that a kindred affection exists among nail feeders; that it is, in fact, much more common among them than the miners. It comes on under similar circumstances, and pursues a similar course, but differs in this particular: the muscles themselves are not involved, but it is confined to the tendons and theca about the wrist. It is much more universal, involving all or nearly all those concerned in pronation and supination. It often leaves permanent disability.—*Ohio Medical Recorder.*

